# Reading dies in complexity: Online news consumers prefer simple writing

**DOI:** 10.1126/sciadv.adn2555

**Published:** 2024-06-05

**Authors:** Hillary C. Shulman, David M. Markowitz, Todd Rogers

**Affiliations:** ^1^ School of Communication, Ohio State University, Columbus, OH 43210, USA.; ^2^ Department of Communication, Michigan State University, East Lansing, MI 48824, USA.; ^3^ John F. Kennedy School of Government, Harvard University, Cambridge, MA 02138, USA.

## Abstract

Over 30,000 field experiments with *The Washington Post* and *Upworthy* showed that readers prefer simpler headlines (e.g., more common words and more readable writing) over more complex ones. A follow-up mechanism experiment showed that readers from the general public paid more attention to, and processed more deeply, the simpler headlines compared to the complex headlines. That is, a signal detection study suggested readers were guided by a simpler-writing heuristic, such that they skipped over relatively complex headlines to focus their attention on the simpler headlines. Notably, a sample of professional writers, including journalists, did not show this pattern, suggesting that those writing the news may read it differently from those consuming it. Simplifying writing can help news outlets compete in the competitive online attention economy, and simple language can make news more approachable to online readers.

## Teaser

News readers engage more with simple writing, suggesting that journalists should write simply to attract attention online.

## INTRODUCTION

How do people select what to read in competitive online news environments? Democratic societies prize a knowledgeable and engaged citizenry, which requires that people educate themselves on the most important and most credible news of the day. In reality, however, even though high-quality news has never been more available, so too is the competition for readers’ attention (*1*). The competition for online attention is fierce. High-quality news must compete for reader attention with misinformation (*2*, *3*) and the proliferation of highly partisan content (*4*–*6*). Against this backdrop, we propose the simpler-writing heuristic as a way of explaining reading behavior in online news environments. Guided by the principle that people are economical with their attention (*7*), we propose that news headlines featuring simpler language will be clicked on, and consequently read, more than news headlines with more complex language. This research sheds light on how people navigate information-rich environments (*8*, *9*), with implications for how news ecosystems can better achieve democratic ideals (*10*).

Evidence across fields and approaches supports our prediction that people prefer simpler news headlines over more complex ones. Experimental evidence suggests simpler texts are rated more positively (*11*) and are engaged with more often (*12*, *13*) than complex texts. Correlational field studies show similar patterns for online engagement in the form of likes and views (*14*), although their implications typically lack strong causal confidence (*15*). Field experiments find that simpler documents (*16*), simpler disclosures (*17*), and simpler applications (*18*) can increase response rates and improve downstream outcomes like showing up for court appearances, signing up for insurance programs, and submitting federal forms. Given the strengths and limitations of this evidence, our understanding of simple writing’s superiority would benefit from large-scale, ecologically valid experimental evidence in the wild.

The simpler-writing heuristic posits that in competitive information environments (e.g., websites with several headlines to select from), simpler writing is more likely to be selected and carefully read for further reading than complex writing. Across nearly 30,000 field experiments conducted with the news sites *The Washington Post* (study set 1) and *Upworthy* (study set 2), we find that readers are more likely to select simply written news headlines relative to complexly written news headlines. Study 3 uses a signal detection task (SDT) (*19*) to provide evidence that general news readers (e.g., people from the general public) more closely read simpler headlines when presented with a set of headlines of varied complexity. In addition to its theoretical implications, the finding that readers engage less deeply with complex writing has important practical implications. Specifically, writing simply can help news creators increase audience engagement even for stories that are themselves complicated.

To this end, in the final study, we test whether professional journalists read news headlines differently from the average, or general public news reader. In other domains, such as law, both professionals and nonprofessionals report disliking complex writing (e.g., “legalese”) (*11*). Given that journalists produce both headlines and the news stories they connect to, understanding whether journalists exhibit similar reading patterns as their readers is of theoretical and practical importance. Thus, in study 4, professional journalists completed the same survey experiment as the general population sample in study 3. Crucially, we found that these professionals did not use the simpler-writing heuristic when reading headlines; they did not select the simpler headlines for further reading or read them more carefully. Apparently, those who write the news read it differently from those who merely consume it. As observed in many other areas, expertise may undermine effective perspective-taking (*20*). This suggests that those who produce high-quality news may not be well suited to effectively present it in competitive online news environments to general audiences. Although bad actors can also use heuristic-based strategies to vie for consumer attention (*21*), the normative goal of this work, which we adopt as well, is to examine whether credible news can benefit from these (verbal) strategies.

This research is supported by the simpler-is-better hypothesis (*14*), which suggests writing that requires less effort to read will tend to be approached, liked (*22*, *23*), and engaged with (*24*, *25*). Therefore, in accordance with this hypothesis, we preregistered the prediction that in study sets 1 and 2, simpler headlines will receive more clicks than more complex ones. We offer a heuristic-based explanation for this, showing that attention is directed toward simple writing and away from complex writing. We test this explanation in studies 3 and 4. We hypothesize that recognition memory will be better for headlines written more simply than for headlines written more complexly. These studies are described below.

## STUDY SET 1: RESULTS

We partnered with *The Washington Post* to obtain all headline experiments run between 3 March 2021, and 18 December 2022 (*N* = 8972 experiments and *N* = 24,044 headlines). From their data and analytics team, we received headline texts, engagement metrics from Chartbeat [e.g., click-through rate (CTR)], and metadata such as the status of the test (e.g., if a winner was found), the author of the headline, and the length of the A/B test. At no point in this project did the authors have access to, nor did we examine, user-level data. Instead, we received headline-level data. Our primary dependent variable was click-through rate (CTR), or “the percentage of visitors who click on a given trial headline” (*26*), which was predicted by the language patterns of each headline. Such internal data from *The Washington Post* was provided to the research team upon reaching a data use agreement, and these tests preceded the authors’ involvement on this project. Our sample size was reduced to 7371 experiments (*n* = 19,926 headlines) after canceled tests by *The Washington Post* were excluded from the dataset. This study was preregistered (https://aspredicted.org/blind.php?x=253_PNN).

**Fig. 1.**
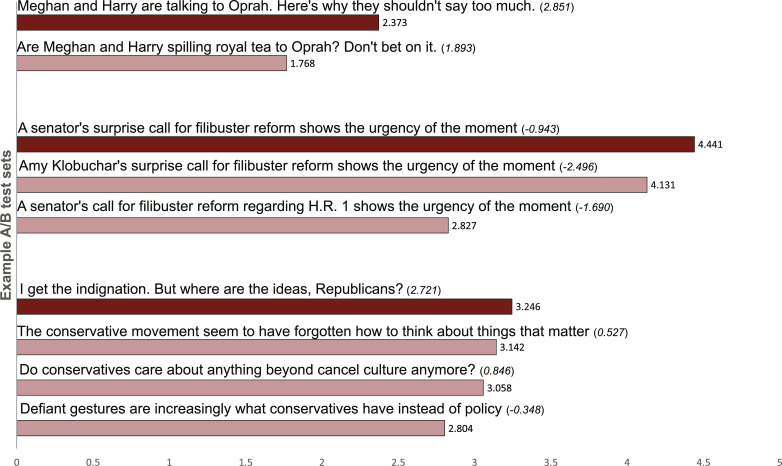
Sample A/B tests and *CTR* from *The Washington Post*. These headline sets were selected to illustrate the range of headlines generated for a given story, and the direction of the simpler-writing heuristic hypothesis. Numbers in italics are scores on the simplicity index with higher scores indicating more simplicity. The dark red bar reflects the simplest version of the headline in a set whereas the light red bars reflect the more complex versions. Bars are presented in order of CTR within each example set.

We used Linguistic Inquiry and Word Count (LIWC) to measure several linguistic properties of the headlines (*27*). LIWC identifies how often each word appears in its internal dictionary of social (e.g., family words), psychological (e.g., emotion terms), and part of speech categories (e.g., prepositions) as a percentage of the total word count. LIWC is a widely used text analysis program for dictionary-based evaluations of language and has been central to many psychology of language studies (*14*, *28*, *29*). The primary independent variable used to assess headline simplicity was a simplicity index we developed. This index was composed of four commonly used markers of linguistic simplicity, including common words, readability, analytic writing, and character count. Our creation of this index, along with details regarding the analysis plan, can be found in the Supplementary Materials. Sample headlines from three experiments, with simplicity index scores and CTRs, are presented in Fig. 1.

The data were evaluated in two ways. First, we took advantage of the A/B test design (described in Materials and Method below) from *The Washington Post* by extracting headlines from within each test that scored highest and lowest on the simplicity index. We also extracted such respective headlines’ CTR. Within each test, we then created difference scores by subtracting the lowest simplicity score from the highest simplicity score, and the associated lowest CTR (e.g., the CTR from the headline with the lowest simplicity score) from the highest CTR (e.g., the CTR from the headline with the highest simplicity score). We performed a simple bivariate correlation on these difference scores, which were natural log transformed due to skewness concerns (see Supplementary Text for more information). The second approach used a linear mixed model to evaluate the link between simplicity and CTR, controlling for within-test dependencies with a random intercept and other fixed effects described in the Supplementary Materials (e.g., the duration of the A/B test).

Consistent with our hypothesis, the difference in the simplicity index was positively associated with the difference in CTR [*r*(7217) = 0.055 and *P* < 0.001; bootstrapped 95% confidence interval (CI) with 5000 replicates (0.049 to 0.095); see fig. S1]. Using the linear mixed model approach, the simplicity index was also positively associated with CTR (*B* = 0.008, SE = 0.001, *t* = 8.84, *P* < 0.001, *R*^2^*m* = 0.092, and *R*^2^*c* = 0.959). Specifically, more common words, a simpler linguistic style, and more readable texts were associated with a higher CTR, although character count was not (see table S1). Together, we found evidence that simple writing is clicked on more than complex writing. As the evidence in Supplementary Text shows, these patterns were robust to content effects as well. Although these effect sizes are small in absolute magnitude, the size of the readership at *The Washington Post* is on the order of tens of millions (*30*). Thus, even a 1% difference could equate to tens of thousands of additional reads (see Discussion for additional commentary on effect sizes). Moreover, these effect sizes are consistent with other studies that have evaluated the impact of language effects on behavior in the wild (*31*).

The results of this first study set suggest that people engage with and click on linguistically simple headlines more than linguistically complex headlines. With these findings in place, we next explore whether these patterns replicate in other types of online writing by examining a similar set of experiments from a storytelling site that focuses on uplifting content, *Upworthy*.

## STUDY SET 1: MATERIALS AND METHODS

Although the research team was not involved in data collection for these experiments, *The Washington Post* provided the team with a general primer about how they approached these experiments. The methodology for these field experiments can be considered an A/B test design. A/B testing refers to a method for comparing two (or more) versions of headline against one another to determine which version performs better [see (*32*) for more information on A/B testing in general]. *The Washington Post* field experiments were collected in collaboration with Chartbeat (*26*). According to this organization’s website, Chartbeat randomly exposed users to one of the trial headlines using the Thompson sampling, or Bayesian bandit, algorithm. Headline exposure was then linked to cookies to ensure that users were exposed to the same headline during the life of the experiment. Some of these headline experiments tested CTR differences across two headline versions (approximately 50% of the tests in our sample), whereas some conducted experiments across more than two headline versions. The language on Chartbeat indicates that this approach constitutes a “live experiment” of headline effectiveness. While we echo this “field experiment” language here, we acknowledge that although these tests include some necessary requirements of experimentation, including manipulation on the independent variable, random assignment, and control (the same story was presented after the headline), there was never a true control group at least for our analytic purposes. After exposure to a trial, a CTR was calculated that assessed the proportion of clicks on a given headline relative to the number of users exposed to that headline version (the percentages provided to the research team). As the CTR for a particular headline began to conclusively favor a headline (with 95% confidence), this headline was determined the winner, the test would complete, and the winning headline would be presented 100% of the time. Notably, for some experiments, a winner was never conclusively determined. In these instances, after 20 min, the test would end and the headline variant with the highest CTR would be chosen (*30*).

Our assessment of linguistic simplicity/complexity considered four main variables of interest: (i) common words, (ii) analytic writing, (iii) readability, and (iv) character count. The rate of common words was measured with LIWC via the dictionary category, which considers the degree to which people use simple, everyday terms (*14*, *29*, *33*, *34*). Analytic writing is a measure of linguistic style composed of seven verbal categories (*35*). Texts that score high on analytic writing tend to be more formal and complex than texts that score low on analytic writing. Readability is a measure of structural complexity and accounts for the number of words per sentence and syllables per word. Using the Flesch Reading Ease metric (*36*, *37*), evidence suggests that texts with more words per sentence and syllables per word are more complex and less readable than text with fewer words per sentence and syllables per word. High scores on the Flesch Reading Ease metric are linguistically simpler (more readable) than low scores. We used the quanteda.textstats package in R to calculate readability (*37*). Last, character count is the raw frequency of characters per headline (including spaces). We evaluated character count instead of word count because word count is a basic component of readability, and this measure would therefore be tautological. All descriptive statistics (see table S2 and fig. S2) and correlations (see table S3) between these variables are provided.

## STUDY SET 2: RESULTS

*Upworthy* data were obtained from prior research (*38*) and consisted of 22,664 unique experiments and 105,551 unique headlines from January 2013 to April 2015. Thus, as with the data from study set 1, the authors were not involved in the creation of the A/B tests and no user-level data were assessed. We used the same text analysis approaches and measures as study set 1, although our dependent variable was slightly different because of engagement metric availability. *Upworthy* provided two engagement metrics, impressions, and clicks. We created a click rate by dividing clicks by impressions (clicks per impression, or CPI), which is conceptually similar to the CTR measure from study set 1. Like in study set 1, we modeled the data by (i) correlating simplicity and CPI difference scores within each A/B test and (ii) as a linear mixed model, controlling for A/B test as a random intercept. This study was preregistered (https://aspredicted.org/blind.php?x=RQH_LML).

The simplicity index was positively associated with CPI [*r*(22,662) = 0.022 and *P* < 0.001; bootstrapped 95% CI with 5,000 replicates (0.021 to 0.054); see fig. S1]. In the linear mixed model, headlines with simpler language received more CPI than headlines with less simple language (*B* = 0.002, SE = 0.001, *t* = 2.45, *P* = 0.014, *R*^2^*m* = 0.00003, and *R*^2^*c* = 0.830). We replicated the common words, analytic writing, and readability effects from study set 1 (table S4), although texts with more characters had higher CPI. Consistent with study set 1, the mixed model results were robust to content effects as well (see the Supplementary Materials for details).

Together, study sets 1 and 2 provided field-based evidence for the simpler-writing heuristic. Next, given the importance of attention in these spaces, we experimentally tested an explanation for the selection effects observed in study sets 1 and 2, namely, that people select headlines based on attention allocation.

## STUDY SET 2: MATERIALS AND METHODS

The *Upworthy* Research Archive, and the methods therein, are discussed at length with the dataset (*38*) that introduced this resource. This archive reports the findings from multiple experiments that broadly examined how various features of news stories (headlines, images, previews, and content) affect a variety of outcomes, including selection. Given that these data were intended for academic use, the method, results, and rigor of the experimental methodology are articulated in the archive. Germane to the current investigation, the unit of analysis for each experiment was a web browsing session. Within this session, users were randomly assigned to receive a particular headline version using the RandomSample method. The outcome was whether participants selected the headline for reading. Editors determined when to conclude a test based on a host of custom calculations that determined the “significance” and relative success of each version of the test. Thus, although details may differ between the data collection or algorithms guiding headline exposure and test administration between study sets 1 and 2, they both sought answers to similar questions regarding which headline version was selected.

Given this similarity, our logic was that if these sites produced similar relationships, these findings support the generalizability of the claims made here. Thus, the goal with study set 2 was not pure replication but rather, generalizability across two different types of news websites that vary in interesting ways. For instance, study set 1 explored headline preferences using data from a legacy news outlet that has a national and international readership, prestige, and influence because millions of people visit this news site every month (*30*). The second study set, while also focused on news reading in the wild, leveraged the expertise of academics to create a news site that would contribute to the general store of knowledge. Thus, the pair of studies offered the opportunity to assess the generalizability of our claims in ecological, and methodologically sophisticated, ways.

*Upworthy* data (the confirmatory package) were obtained from prior research (*37*) and consisted of 22,664 experiments and 105,551 headlines from January 2013 to April 2015. We used the same text analysis approaches and measures as study set 1, although our dependent variable was slightly different because of engagement metric availability. *Upworthy* provided two engagement metrics, impressions and clicks. We created a click rate by dividing clicks by impressions (CPI), which is conceptually similar to the CTR measure from study set 1. CPI values were re-expressed using the formula ln(*Y* + 0.001) out of skewness concerns. As with study set 1, we provide all descriptive (table S2 and fig. S3) and correlational information (table S3) about these variables.

## STUDY 3: RESULTS

This survey experiment had two aims. The first was to replicate in a more controlled environment whether a simple version of a headline chosen from *The Washington Post* received more clicks than a complex version of the same headline. Thus, participants read 10 headlines and indicated which headline they would be likely to select if they were reading the news. Information about the construction of these headlines is provided in the Supplementary Materials (table S5). The second aim was to understand the underlying cognitive process driving people to select simpler headlines. For this, we used a 24-item SDT paradigm (*19*), which is designed to assess recognition memory, or here, attention. The SDT was designed to assess whether people allocated more attention to simple texts relative to complex texts using a measure of sensitivity (*d*′). The higher the sensitivity score, the more attention and retention paid to the headline. This study was preregistered (https://aspredicted.org/7NV_PZR) and an a priori power analysis ensured that we had enough participants to detect a small effect at 95% power. Data, syntax, and output can be found on our Open Science Framework (https://osf.io/nwsgf/?view_only=55f56aec468c4d8699787b416b7afdee) page.

**Fig. 2.**
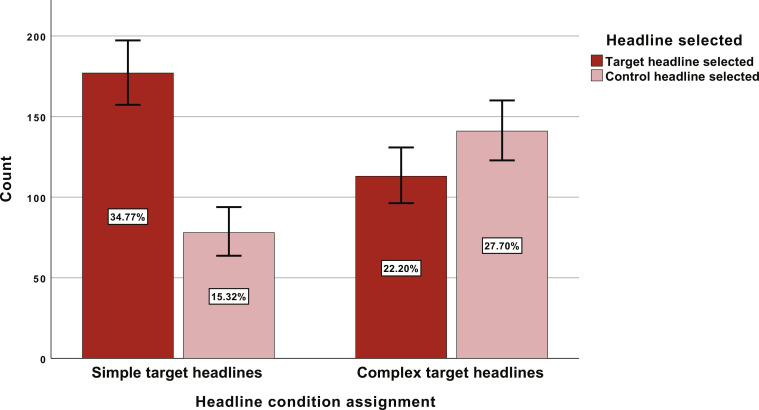
Headline selections based on experimental condition. Error bars reflect 95% CIs surrounding the estimates presented. These estimates reflect the percentage of participants who selected a particular headline type.

The results suggested that participants were significantly more likely to select simple headlines compared to more complex ones [χ^2^(1, *N* = 524) = 32.25, *P* < 0.001, and odds ratio = 2.83]. Specifically, when target headlines were written simply, they were selected more (*n* = 177, 34.8%) than the control headlines (*n* = 78, 15.3%). Alternatively, when the target headlines were written using complex language, they were selected less (*n* = 113, 22.2%) than the control headlines (*n* = 141, 27.7%; see Fig. 2).

The SDT outcome was also consistent with our hypothesis, as participants in the simple headline condition (*M* = 1.23 and SD = 0.81) demonstrated significantly better sensitivity compared to those in the complex headline condition [*M* = 0.80 and SD = 0.77; *t*(483) = 6.01, *P* < 0.001, and Cohen’s *d* = 0.55]. This means that complex headlines were less likely to be selected, and that minutes later, the phrases in complex headlines were less likely to be recognized compared to simple headlines.

## STUDY 3: MATERIALS AND METHODS

Participants in this survey experiment (*N* = 524) were recruited from Amazon Mechanical Turk via CloudResearch (*39*) from 2 to 8 May 2023. After being provided with screening measures to ensure that participants could pass a CAPTCHA and were at least 18 years old, participants were presented with an approved consent form (no. 2023E0422) from the lead author’s institution. This sample identified as 53.8% male, 45.2% female, and 0.6% nonbinary, with 1% of participants’ data either missing (*n* = 3) or preferred not to say (*n* = 2). The average age of the sample was 41.70 years old (SD = 12.03 and range = 20 to 77) and 76.4% of participants identified as white, 10.8% Black, 6.8% Asian, 1% American Indian or Alaska Native, and 2.8% other or multiracial. To obtain higher-quality data, participants were eligible to participate if they could pass a CAPTCHA, could respond to an open-ended prompt, and had at least a 95% completion rating on at least 500 human intelligence tasks. Participants were compensated $2.00 for their time.

To create the SDT measure, participants were presented with a three-word phrase and asked whether this phrase appeared (coded as 1) or did not appear (coded as 0) in the set of headlines they viewed. A “hit” was an instance in which participants accurately reported that they saw a phrase (true positive) or accurately reported that they did not see a phrase (true negative). Alternatively, “foils” were instances where participants incorrectly reported that they saw a phrase when they did not (false positive) or alternatively, when they stated that they did not see a phrase when they did (false negative).

## STUDY 4: RESULTS

Study 4 included the same headline selection and SDT as study 3. The only difference was that this sample consisted of professional journalists and writers. Because this sample was different and the recruitment efforts were different, a new institutional review board protocol was used and approved (no. 2023E0883) by the lead author’s institution. This study was preregistered (https://aspredicted.org/DQQ_Y6T) using the same hypotheses as study 3 and an a priori power analysis based on the study 3 effect size ensured enough participants were recruited.

**Fig. 3.**
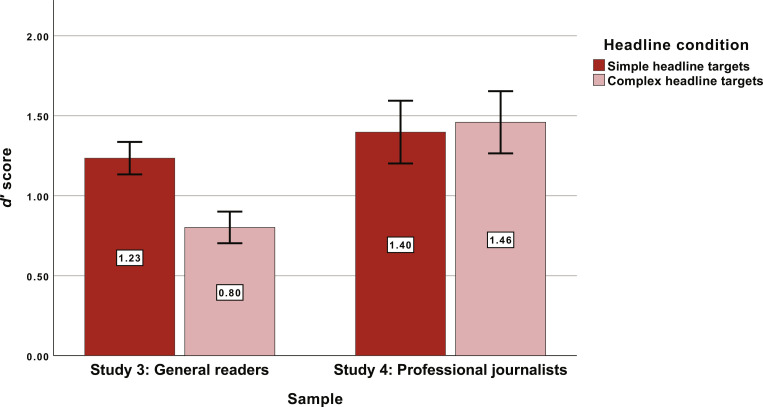
Differences in task performance between studies 3 and 4. Error bars reflect 95% CIs surrounding the estimates. These sample estimates reflect data from crowdsourced workers in study 3 and the sample of professional writers in study 4. For more detailed occupational demographics for study 4, see the Supplementary Materials.

The results from study 4 produced a notable departure from studies 1 to 3. First, the results of the headline selection task were not significant [χ^2^(1, *N* = 225) = 0.36 and *P* = 0.549]. Second, the results of the SDT were also not significant [*t*(165) = −0.44, *P* = 0.660, and Cohen’s *d* = 0.07]. Together, these null findings suggest that, for journalists, headline simplicity does not affect selection, attention, or memory. One notable finding from the SDT was just how well journalists performed. This value of sensitivity (*M* = 1.43 and SD = 0.91) was significantly higher than the sensitivity observed in the general population sample [*M* = 1.02 and SD = 0.82; *t*(523) = 3.01, *P* < 0.01, and Cohen’s *d* = 0.47], which suggests journalists appear to vigilantly read and remember what they read (see Fig. 3).

One final piece of evidence provides support for the idea that journalists read differently from the general population sample from study 3. Journalists were presented with six headline pairs from *The Washington Post* and were asked if they could correctly identify the winning headline. Here, journalists performed no better than chance [50% accuracy, *M* = 3.09, SD = 1.36, *t*(146) = 0.79, *P* = 0.432, and *d* = 0.07], suggesting a disconnect between what journalists think audiences will read and what they actually do. We consider this disconnect, along with the methodological, theoretical, and practical implications of these reading habits in General Discussion.

## STUDY 4: MATERIALS AND METHODS

Participants in this survey experiment (*N* = 249) were recruited from a list of participants enrolled in a webinar on effective writing. This webinar included a presentation by one of the authors of this article. Before the webinar, attendees were asked to participate in a brief survey relevant to the presentation. They were told that the results of this survey would be of interest to them and would be shared during the presentation. The attendees of this webinar were a particularly interesting sample because they all identified as professional writers, including mostly current and former journalists (average of 13.86 years of experience, SD = 14.60, and *n* = 122, 47%), but also educators, communication directors, and government employees (see table S6 for occupational demographics). This sample identified as 32.5% male, 63.9% female, and 0.8% nonbinary, with 2.8% of participants’ data either missing (*n* = 4) or preferred not to say (*n* = 3). The average age of the sample was 52.38 years old (SD = 15.38 and range = 18 to 93) and 73.1% of participants identified as white, 5.6% Black, 8.4% Asian, 0.8% American Indian, Alaska Native, or Pacific Islander, and 8.4% other or multiracial (2.8%).

## GENERAL DISCUSSION

Thousands of field experiments across traditional (i.e., *The Washington Post*) and nontraditional news sites (i.e., *Upworthy*) showed that news readers are more likely to click on and engage with simple headlines than complex ones. General readers were also more likely to recognize phrases from the simpler headlines than from complex headlines. These results are consistent with our theory that in crowded information environments, people are guided by a simpler-writing heuristic: People use the simplicity/complexity of the writing they encounter as a cue for what writing they will engage with and attend to.

There are several important takeaways from this package of studies. First, the consistencies across the first three studies suggest that, overwhelmingly, general readers are economical with their attention and that the simpler-writing heuristic provides a useful explanation for how people decide what to read online. Practically, this finding implies that small-scale efforts aimed at increasing the simplicity or fluency of language can increase the attention of casual readers. Second, the findings observed from the journalists’ sample suggests that journalists may exhibit a different, and more thorough, approach to news reading. This thoroughness was evident in their news selection (no preference for the simpler titles) and in their high level of recognition memory across headlines. Notably, this observation presents a departure from other research that has found in professions like law, lawyers, and nonlawyers alike report a distaste for legalese (*11*).

There are a few potential explanations for the disconnect between the general readers’ sample and the professional writer sample that merit further exploration. The first is methodological. It is possible that these two study samples approached the task differently. Specifically, journalists might have felt motivated to perform better because their performance reflected on their professional identity. General news readers, by contrast, might have approached the task more casually and as a result, underperformed relative to journalists. Although prior research has similarly found that motivation reduces the impact of heuristics, such as language complexity on topic engagement (*40*–*42*), this explanation does not quite square with all of the evidence obtained in our work. For instance, in the follow-up A/B test, where journalists’ motivation should be quite high (“Can you guess the winning headline?”), journalists performed no better than chance at guessing *The Washington Post* headline that received the most clicks. Thus, when journalists were directly asked to perspective take about consumers’ preferences, they were unable to do so accurately. Second, although demand characteristics may have been high for the journalists’ sample, the benefit of using signal detection to assess attention allocation is that it uses a behavioral measure that, unlike self-report, is less susceptible to demand characteristics. Thus, even though there are some methodological differences between studies 3 and 4 due to sample recruitment, it is hard to imagine how these differences explain the entirety of the effects that we observed.

The different reading approaches of those who create the news and those who consume it may lead to consequential blind spots. The possibility that journalists are more motivated to carefully read and process the news, relative to general news readers, may suggest a disconnect between what journalists think audiences want to read, and what audiences actually read. Future work investigating this potential disconnect is important theoretically, to illuminate boundary conditions for the simpler-writing heuristic and, practically, to help news organizations understand where they can improve.

When looking at the findings across study sets 1 and 2, we note that the effect sizes obtained for simple writing are consistent with other language-based field experiments in the psychology of language area. For example, a paper by Kramer *et al.* (*31*) found that modifying rates of emotion in one’s Facebook newsfeed changed rates of emotion in their subsequent posts, with a very small but systematic effect size (Cohen’s *d* = 0.02, equivalent to *r* = 0.01). On the basis of Facebook’s population size, language-based effects even at this magnitude can still lead to a nontrivial downstream behavioral impact at scale. The effect sizes that we observe are consistent with prior work, and the scale of our news sources similarly suggests real-world consequences. To illustrate, on the basis of audience traffic data from *The Washington Post* (*30*), there was an average of about 70 million digital unique visitors to the site per month during the time period in question (March 2021 to December 2022). If we assume that each visitor reads three stories, small percentage differences at this scale matter greatly: A CTR difference of 0.10% (2.1% versus 2.0%) still equates to a difference of over 200,000 readers of the simpler stories based on headline simplicity alone.

Together, this work highlights the benefits of language simplicity as one of many elements that can increase demand for and attention to credible news. While many features can affect attention and selection of news headlines (*43*), one benefit of linguistic simplicity is its ease of implementation, even for otherwise complex stories. In online spaces where less credible (*21*) and highly polarized sources (*4*) already tend to use simpler writing, we suggest that the simpler-writing heuristic can increase demand for credible journalism in a competitive attention economy.

## Supplementary Materials

### This PDF file includes:

Supplementary Text Figs. S1 to S3 Tables S1 to S8 References
