# Supplementary material for: Reading dies in complexity: Online news consumers prefer simple writing
Source: Sci Adv. 2024 Jun 5;10(23):eadn2555. doi: 10.1126/sciadv.adn2555 (PMC11152133; doi:10.1126/sciadv.adn2555)
Supplement: Supplementary file 1 — Supplementary Text Figs. S1 to S3 Tables S1 to S8 References [file sciadv.adn2555_sm.pdf]

Science Advances
AAAS

Supplementary Materials for

Reading dies in complexity: Online news consumers prefer simple writing

Hillary C. Shulman *et al.*

Corresponding author: Hillary C. Shulman, shulman.36@osu.edu.

Sci. Adv. **10**, eadn2555 (2024)
DOI: 10.1126/sciadv.adn2555

This PDF file includes:

Supplementary Text
Figs. S1 to S3
Tables S1 to S8
References

This document provides additional details for the methodology, data analysis, and results across all four studies. In addition to this document, data, syntax, and output can be located at our open science framework site. Although the data from Studies 2-4 are available on this site, the data use agreement signed between the authors of this study and *The Washington Post* prevented us from being able to share their internal data with people beyond the research team. If there are any questions about this, the authors would be happy to elaborate upon the details of this agreement.

Study Set 1: Additional Information

Analytic Plan

Linear mixed models (*44*, *45*), with random intercepts for the A/B test and author of the headline, evaluated the link between language patterns and *CTR*. We also included two fixed effect controls: (a) the status of an A/B test (1 = winner found, test not stopped; 0 = test stopped with winner found), and (b) the duration of the A/B test in seconds, which helped to account for the idea that a higher *CTR* might be due to a test being available for more time.

We took a layered approach to evaluate how language patterns were associated with *CTR*. First, we standardized (z-scored) each measure and applied the following formula to develop a *simplicity index*: common words + readability – analytic writing – character count. High scores on this measure indicate simpler linguistic patterns than low scores, and the *simplicity index* allowed us to obtain a global understanding of how words link to *CTR*. Second, separate models for each language dimension were constructed, which helped to identify the most robust linguistic links to *CTR*. Out of skewness concerns, we re-expressed *CTR* by natural log-transforming each value and adding a constant. The formula for re-expression was $ln(Y + 0.10)$ for the mixed model calculations.

Results

We provide a visual description in fig. S1 of the minimum and maximum analyses reported in the main text for the *simplicity index*. The bivariate relationship between *simplicity index* items and click-through rate based on the minimum and maximum analyses were as follows: for common words ($r = .021$, $p = .080$), analytic writing ($r = -.061$, $p < .001$), readability ($r = .024$, $p = .04$), and character count ($r = -.042$, $p < .001$). We also provide tables of the linear mixed model results for transparency (table S1).

Alternative Explanations

One alternative, post-hoc, explanation for our results is that content effects might be driving headline selection above and beyond linguistic simplicity. To address this possibility, we deviated from our preregistered analysis plan and automatically extracted dominant themes from the headlines using the Meaning Extraction Method (*46*, *47*). The aims and interests of the Meaning Extraction Method are conceptually similar to other common topic modeling approaches, namely Latent Dirichlet allocation (LDA) (*48*). Indeed, prior work has compared outputs of the Meaning Extraction Method and LDA, finding converging and similar results in thematic extraction (*49*). This approach removes style words and low base-rate words from the headlines, and using Principal Component Analysis with varimax rotation, identifies how content words (e.g., nouns, verbs) cluster statistically. This method produced five systematic themes (see themes and their component loadings in table S7): headlines related to (1) “things to know today” (a series from *The Washington Post*), (2) the Ukraine War, (3) climate change, (4) the White House, and (5) COVID-19. Themes were saved as regression weights for future use in linear mixed model calculations. That is, based on the presence or absence of certain content words, headlines were given a standardized score suggesting how much each headline reflected each theme (high scores indicate headlines that contained more of each respective theme than

low scores). Note that the output for the Meaning Extraction Method is a binary matrix representing the presence or absence of a particular term across each headline. For example, if the word *Putin* appeared in Headline A, it would receive a score of 1. If the word *Putin* did not appear in Headline B, it would receive a score of 0. This binary output (0 or 1) follows best practices for the Meaning Extraction Method. For words to be retained in this analysis, they must have appeared in at least 1% of the headlines in each dataset.

The relationship between simplicity and *CTR* was statistically significant after accounting for each theme as a fixed effect in the prior linear mixed model calculation (B = 0.008, SE = 0.001, t = 8.81, p < .001). Headlines that related more to the White House (p < .001), less to climate change (p = .013), and less to COVID-19 (p = .047) tended to receive a higher click-through-rate. Together, this evidence suggests our simplicity effects are robust to content and other covariates using a legacy and traditional journalistic outlet.

Study Set 2: Additional Information

Analytic Plan

We first used a linear mixed model to associate the *simplicity index* with *CPI*, followed by separate models for each language dimension of interest. Each model contained a random intercept for A/B test since headlines within each test were not independent.

Results

We provide a visual description of the minimum and maximum analyses reported in the main text for the *simplicity index* (see fig. S1, right panel). The bivariate relationship between simplicity index items and clicks-per-impression based on the minimum and maximum analyses were as follows: for common words (r = .152, p < .001), analytic writing (r = -.037, p < .001), readability (r = .023, p < .001), and character count (r = .089, p < .001). We also provide tables of the linear mixed model results for transparency (table S4).

Alternative Explanations

Consistent with Study set 1, we deviated from our preregistered analysis plan to evaluate the degree to which our simplicity effects were robust to content. We extracted five dominant themes from the headlines using the Meaning Extraction Method approach described earlier. Reliable and systematic themes related to: (1) race/ethnicity, (2) question-asking, (3) gender, (4) watching videos, and (5) societal problems. Themes were saved as regression weights.

In a linear mixed model controlling for such themes as fixed effects, the evidence suggested simplicity remained positively associated with *CPI* (B = 0.002, SE = 0.001, t = 3.16, p = .002). Themes related to race/ethnicity, question-asking, gender, and watching videos were positively associated with *CPI* (p's < .001), and the theme of societal problems was negatively associated with *CPI* (p = .003). Again, with a new study using a vastly different and non-traditional journalistic outlet, the evidence suggests linguistic simplicity is associated with engagement above and beyond content effects (see table S8).

Commentary on Statistical Re-Expression

Readers will notice several analyses in this paper used variables that were re-expressed (natural log-transformed). This was purposeful, and followed best practices upon the identification that certain variables were indeed skewed.

The specific re-expression formulae were dependent on the statistical test under consideration. For example, Study sets 1 and 2 had two main analyses: (1) correlational, and (2) those involving the linear mixed models. The correlational analyses added a constant to each variable (value = 1), which when presented in a scatterplot, suggested this re-expression

represented the data best and retained the greatest number of A/B tests in the analyses. Other re-expressions, including *ln*(X + .1) or *ln*(X + .01), produced conceptually equivalent results (and larger effect sizes), but substantially reduced the number of tests under consideration due to the presence of impossible values (dividing by zero). We therefore decided to use the formula with the constant equal to 1 for transparency and generalizability.

In the linear mixed models, re-expression of the dependent variables was based on the authors' interpretation of Q-Q plots and familiarity with similar data structure. We offer this commentary in the spirit of transparency.

Study 3: Additional Information

Procedure

If participants consented to participate, they were randomly assigned via Qualtrics software to one of two experimental conditions that presented either a simple (n = 258) or complex (n = 266) set of 10 news headlines. Six of these headlines were directly taken from *The Washington Post* (control headlines) and contained higher than average complexity. The other four headlines (target headlines) were modified versions of original *Washington Post* headlines. To make these sets, the authors first selected two *Washington Post* headlines that were in the top 1% of the simple headlines provided, and then did the same with two headlines that were in the bottom 3% percent. With these original headlines as templates, a thesaurus was used to replace original words with their more complex (or simple) counterparts. This approach has been taken in other research using a language complexity manipulation (*22*, *50*). When these headline pairings were created, care was taken to keep headline word counts as consistent as possible (within two words of one another, see table S5). This approach allowed us to vary the complexity of headline language without modifying the substance, or form, of the original headline.

During this headline task, participants were provided with the following prompt: "On the next page you will view 10 news headlines. Imagine that you were browsing the home screen of a newspaper on your computer or reading a newspaper at home. We are interested in knowing which headline you would be likely to click on. When you are ready to proceed to the next page, please click the advance button below." Importantly, participants were not informed beforehand that they were going to be asked about these headlines again.

After participants selected the headline they would be likely to read, they went on to answer a series of filler items. These items asked general questions about news reading and interest in the news. The purpose of these questions was to provide a distractor task in between the headline selection task and the signal detection task described below. After the signal detection task, participants provided their demographic information. This demographic information is not provided in the data available on the OSF site in order to protect participant identity. This information can be made available, however, upon request. In total, this survey took 9.30 minutes to complete (SD = 11.63 minutes, Median = 6.70 minutes).

Signal Detection Task

The purpose of this section is to provide more detail regarding the signal detection task. First, across both the simple and complex language conditions participants filled out the same 24 item set. Of these 24 items, 12 were phrases that were coded as either a hit or a foil depending on condition assignment (e.g., "should work out" vs. "prudent to exercise"). Then six items were hits common to both conditions, and six items were foils common to both conditions. The instructions preceding this task stated "We are now interested in what you remember from the headlines you read. In the following section, you will see 2-3 word phrases that may or may not have appeared in the headlines you read earlier in the study. After reading each phrase, please indicate either: "Yes" – meaning you saw this phrase in a headline, or "No" – meaning you did

not see this phrase in a headline." To better ensure attention across the entirety of these headlines, care was taken to ensure that different parts of each headline (beginning, middle, end phrases) were equally represented. Some examples of these three-word phrases include: "causes union talks", "make cocaine legal", "laborious endeavors", "in the sky", and "has new idea". Notably, phrase placement within a headline did not impact signal detection performance.

Outcomes

The first outcome was referred to in the main article as *headline selection*. This outcome reflects the article chosen, or clicked on, by participants. If a participant chose one of our manipulated, or "target," headlines this response was coded as 1, and if participants selected one of our control headlines this response was coded as 0.

To measure recognition memory using a signal detection task, a "sensitivity score" (*12*) was calculated by measuring the distance (in standard deviations) between the hit and foil distributions, a measure known as *d'* (d-prime). Thus, sensitivity (*d'*) can be conceptually interpreted as participants' ability to discern hits (i.e., signals) from foils (i.e., noise), in which higher scores reflect better sensitivity. We opted for this behavioral measure of attention because it is less prone to demand characteristics than self-report measures of attention (see General Discussion in the main text for more information and commentary on this matter).

Robustness Check

Similar to the post-hoc analyses run for Study sets 1 and 2, for Study 3 we ran an exploratory post-hoc analysis to assess whether the relationship between condition assignment and signal detection task performance was maintained when accounting for crowd workers' news reading habits and level of education. Specifically, a regression was run using *condition assignment*, *news interest*, *news reading frequency*, and *level of education* as predictors, with *d'* scores as the dependent variable. It was found that the relationship between condition assignment and signal detection task performance remained significant, and in the expected direction, $B = -0.45$, $SE = 0.08$, $t = -6.03$, $p < .001$, even when controlling for news habits and level of education. Thus, language simplicity facilitates attention above and beyond what would be expected by daily news reading habits or education.

Limitations

The goal of this research was to understand the reading habits of general news readers. To obtain this information, we relied on crowd workers who were compensated for their participation. Although using crowdsourced workers has become commonplace in the social sciences, there are known issues regarding the representativeness of this sample and potentially concerns about data quality(*51*). Thus, we want to acknowledge these limitations.

Study 4: Additional Information

Participants

Because the occupational demographics of this sample were of interest to Study 4, we provide a table of the professional characteristics of this sample. Despite the availability of some professional information, given that this study was voluntary and did not provide any monetary incentives, it is not surprising that a lot of these data were missing. Nevertheless, we report the data we have in table S6. We also want to note that some of these data are excluded from the datafile available on OSF to maintain participants' anonymity. If anyone is interested in more details about this information they are welcome to reach out to the authors of this study.

Procedure

This survey experiment was almost identical to the survey experiment described in Study 3 including all task instructions. After providing consent, participants were presented with the

same headline selection task described in Study 3. There were two small differences between these survey experiments. The first was the filler task. While in Study 3 the filler items inquired about general news reading, in this study questions were asked about participants' professional experiences given the purpose of this study. The second difference was, following the same signal detection task as Study 3, these participants were provided with a short, six item test. The instructions for this test read, "*The Washington Post* has conducted lots of A/B tests to find the best headlines for stories. In the next two pages, we provide some of those A/B tests. For each pair of headlines, please select the one that you think had a higher click-rate?" Participants then viewed six pairs of original headlines from *The Washington Post* to see whether journalists could intuit which headlines were successful. Performance on this test was coded as a 0 for an incorrect answer, and 1 for a correct answer, and scores were summed to create the accuracy scale reported in the paper (range 0 - 6). In total, this task took approximately 11.68 minutes to complete (*SD* = 23.65 minutes, Median = 6.73 minutes).

Outcomes

The same data analysis as in Study 3 were also used for Study 4. The primary outcomes were headline selection (targets versus controls) and the sensitivity score on the SDT task.

Supplemental Figures and Tables

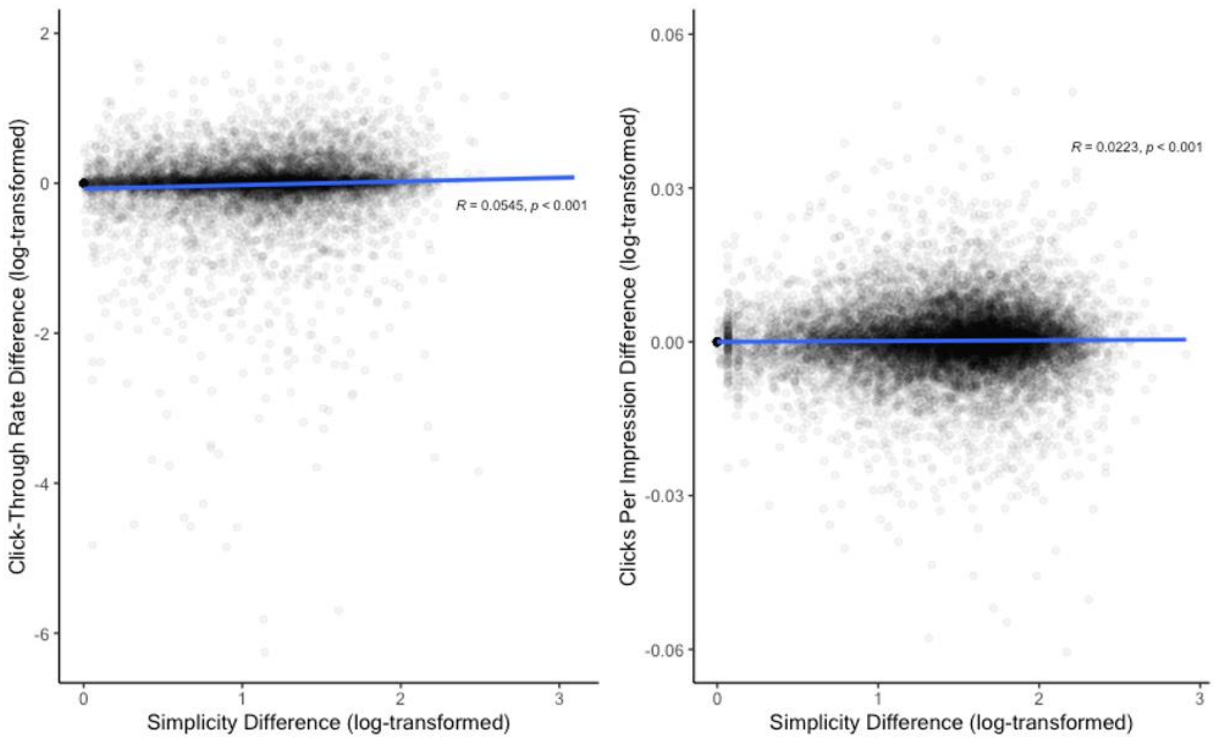

fig. S1. Bivariate relationship between simplicity and *CTR* in *The Washington Post* (left panel) and *CPI* in the *Upworthy* sample (right panel).

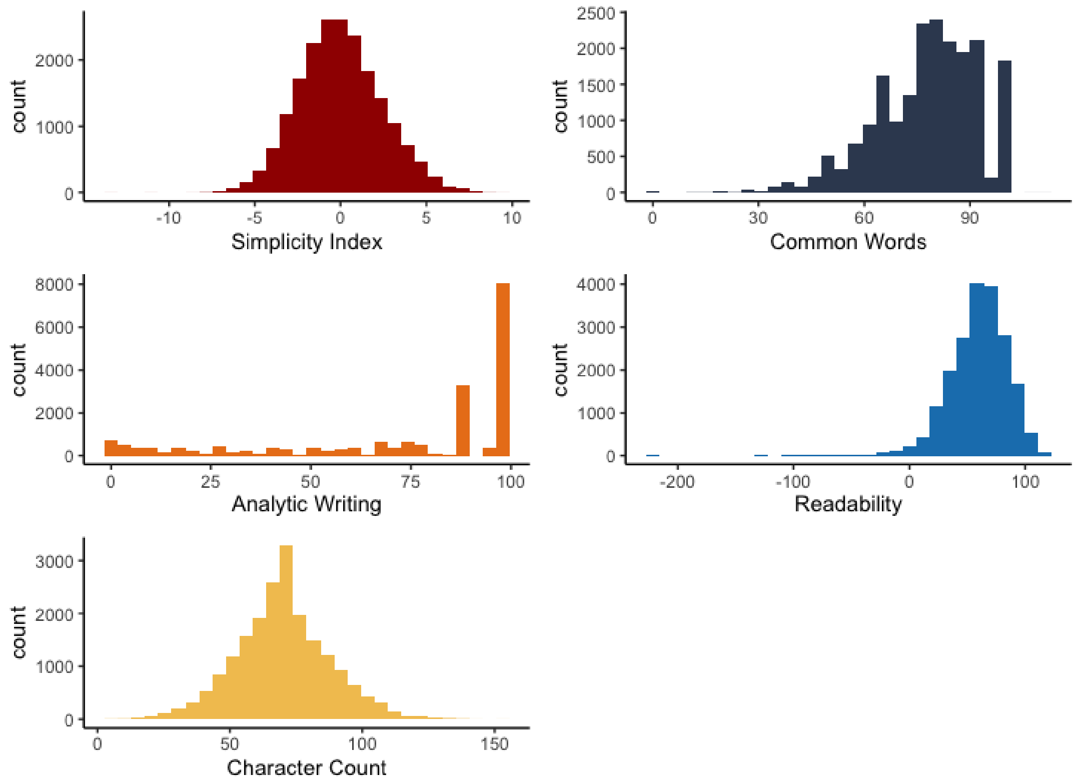

fig. S2. Histograms for simplicity variables in *The Washington Post* sample.

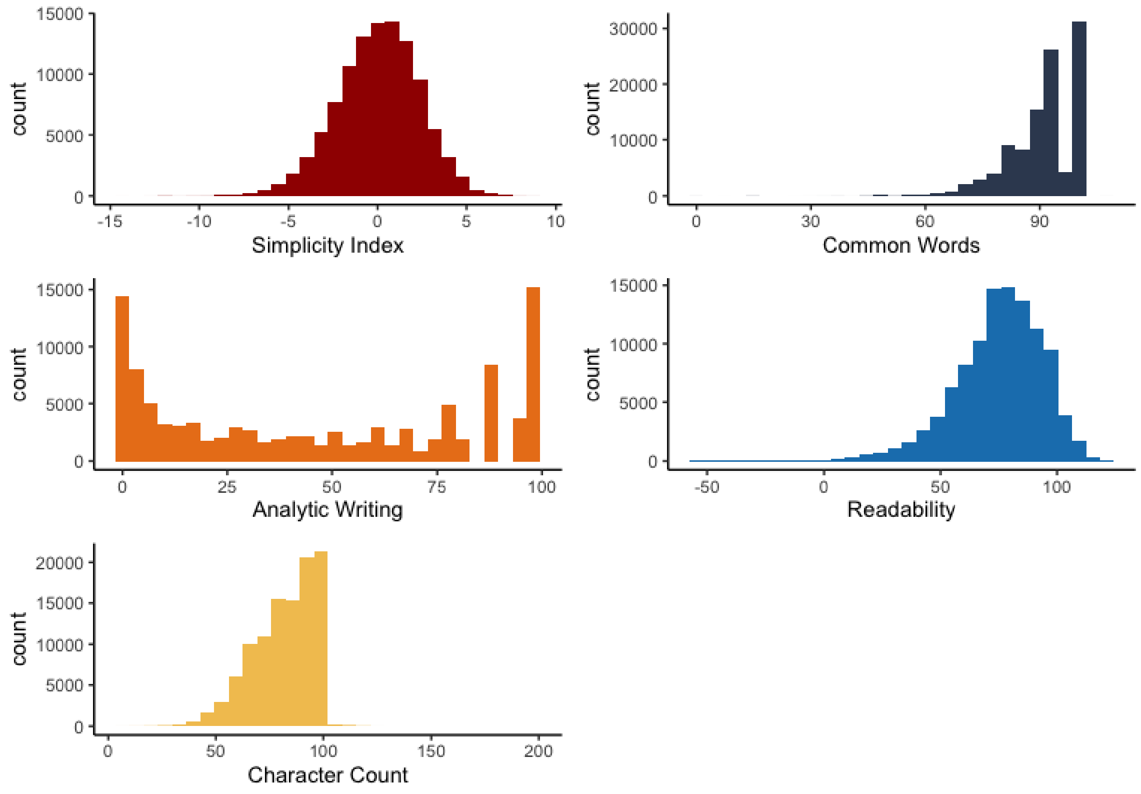

fig. S3. Histograms for simplicity variables in the *Upworthy* sample.

table S1. The multivariate relationship between simplicity variables on *CTR* in *The Washington Post.*

Fixed effects	*B*	*SE*	*df*	*t*	*p*
Intercept	-0.127	0.065	47.91	-1.95	0.0568
**Simplicity index**	0.008	0.001	13664.77	8.84	< .001
Status: Winner found	-0.091	0.022	7357.05	-4.10	< .001
Test duration (z-scored)	-0.271	0.010	7310.46	-27.25	< .001
Random intercepts	*n*	σ^2	*SD*		
Test	7371	0.554	0.744		
Author	47	0.161	0.401		

Fixed effects	*B*	*SE*	*df*	*t*	*p*
Intercept	-0.185	0.066	51.392	-2.79	.007
**Common words**	0.001	0.000	13839.297	4.29	< .001
Status: Winner found	-0.090	0.022	7355.424	-4.07	< .001
Test duration (z-scored)	-0.271	0.010	7309.059	-27.26	< .001
Random intercepts	*n*	σ^2	*SD*		
Test	7371	0.554	0.744		
Author	47	0.160	0.400		

Fixed effects	*B*	*SE*	*df*	*t*	*p*
Intercept	-0.0967	0.065	48.448	-1.48	0.145
**Analytic writing**	-0.0005	0.000	13270.082	-7.51	< .001
Status: Winner found	-0.0903	0.022	7357.282	-4.08	< .001
Test duration (z-scored)	-0.2704	0.010	7311.090	-27.25	< .001
Random intercepts	*n*	σ^2	*SD*	-	-
Test	7371	0.553	0.744		
Author	47	0.160	0.400	-	-

Fixed effects	*B*	*SE*	*df*	*t*	*p*
Intercept	-0.164	0.065	48.556	-2.51	0.0156
**Readability**	0.001	0.000	13554.857	6.43	< .001
Status: Winner found	-0.091	0.022	7357.331	-4.08	< .001
Test duration (z-scored)	-0.270	0.010	7310.701	-27.25	< .001
Random intercepts	*n*	σ^2	*SD*		-
Test	7371	0.554	0.744		

Author	47	0.161	0.401		-

Fixed effects	*B*	*SE*	*df*	*t*	*p*
Intercept	-0.1218	0.0657	49.510	-1.85	0.0699
**Character count**	-0.0001	0.0001	13254.010	-1.22	0.223
Status: Winner found	-0.0903	0.0222	7357.589	-4.07	< .001
Test duration (z-scored)	-0.2706	0.0099	7310.759	-27.27	< .001
Random intercepts	*n*	σ^2	*SD*	-	-
Test	7371	0.553	0.744		
Author	47	0.160	0.401	-	-

table S2. Descriptive statistics across simplicity variables in Study sets 1 and 2. For the simplicity index creation, the four variables underlying the simplicity index were standardized. *N* = 19,926 for Study set 1 and *N* = 105,551 for Study set 2.

	Study set 1			Study set 2		
Variable	*M*	*SD*	Median	*M*	*SD*	Median
Simplicity index	0.00	2.40	-0.11	0.00	2.32	0.12
Common words (%)	77.81	14.52	80.00	90.85	9.01	92.12
Analytic writing	73.11	32.48	89.52	46.60	37.13	43.40
Readability	59.33	24.58	61.33	75.05	18.43	76.97
Character count	69.89	17.52	70.00	81.82	14.48	84.00

table S3. Correlations between simplicity variables in Study sets 1 and 2. Correlations between all variables that comprise the *simplicity index*. All correlations are Pearson correlations and based on raw values. *** $p < .001$

Study set 1 (*The Washington Post*)				
Pearson's *r*	Common words	Analytic writing	Readability	Character count
Common words	--			
Analytic writing	-.214***	--		
Readability	.300***	-.192***	--	
Character count	.032***	.068***	-.134***	--

Study set 2 (*Upworthy*)				
Pearson's *r*	Common words	Analytic writing	Readability	Character count
Common words	--			
Analytic writing	-.232***	--		
Readability	.320***	-.192***	--	
Character count	.070***	-.040***	-.061***	--

table S4. The multivariate relationship between simplicity variables on *CPI* in the *Upworthy* sample.

Fixed effects	*B*	*SE*	*df*	*t*	*p*
Intercept	-4.282	0.004	22600.415	-1009.17	< .001
**Simplicity index**	0.002	0.001	98851.820	2.45	.014
Random intercepts	*n*	σ^2	*SD*		
Test	22664	0.390	0.624		

Fixed effects	*B*	*SE*	*df*	*t*	*p*
Intercept	-4.531	0.017	104046.509	-269.17	< .001
**Common words**	0.003	0.000	98937.169	15.28	< .001
Random intercepts	*n*	σ^2	*SD*		
Test	22664	0.391	0.625		

Fixed effects	*B*	*SE*	*df*	*t*	*p*
Intercept	-4.2678	0.005	31165.131	-917.93	< .001
**Analytic writing**	-0.0003	0.000	96455.238	-7.60	< .001
Random intercepts	*n*	σ^2	*SD*		
Test	22664	0.390	0.625		

Fixed effects	*B*	*SE*	*df*	*t*	*p*
Intercept	-4.2994	0.008	91137.982	-563.62	< .001
**Readability**	0.0002	0.000	97791.322	2.72	.007
Random intercepts	*n*	σ^2	*SD*		
Test	22664	0.390	0.624		

Fixed effects	*B*	*SE*	*df*	*t*	*p*
Intercept	-4.454	0.010	103645.525	-459.46	< .001
**Character count**	0.002	0.000	97705.878	19.68	< .001
Random intercepts	*n*	σ^2	*SD*	-	-
Test	22664	0.388	0.623	-	-

table S5. Experimental headline selection task. An asterisk denotes original headline language from *The Washington Post.* The control headlines were all original headlines.

Headline type	Headline text
**Target Headlines**	
Simple	You should work out on your next long flight. Here's how*
Complex	It's prudent to exercise on your future extended flight. An explainer
Simple	Why it feels good to do hard things*
Complex	Explaining the hedonic impact of undertaking laborious endeavors
Simple	Columbia has a new idea: Make cocaine legal
Complex	Columbia contemplates radical experiment: Decriminalize cocaine*
Simple	Amazon's worker monitoring causes union talks
Complex	Amazon's employee surveillance fuels unionization efforts*
**Control Headlines**	
Mt. Kilimanjaro gets high-speed internet, a bid by Tanzania to boost tourism	
Start-up news site Semafor ran Chevron sponsorship alongside climate coverage	
Build-to-rent developments exacerbate long-simmering inequalities in housing, critics say	
White House summons China ambassador amid widening diplomatic crisis over Pelosi Taiwan trip	
Is Biden impervious to impressionists?	
Anxiety, resentment fester at Facebook as executives outline higher expectations	

table S6. Characteristics of sample recruited from webinar. For these items, participants were allowed to select multiple options.

**Which of the following best describes your job?**	*n* (%)
Reporter	26 (10.4%)
Section Editor	5 (2.0%)
Copy Editor	7 (2.8%)
Managing Editor	12 (4.8%)
Editor in Chief	11 (4.4%)
Digital Editor	10 (4.0%)
Other	81 (61.0%)
Missing	97 (39%)
**What is the circulation of your publication?**	
Under 10K	60 (24.1%)
10K-25K	15 (6.0%)
25K-50K	8 (6.0%)
50K-100K	7 (2.8%)
100K-300K	8 (3.2%)
200K+	17 (6.8%)
Missing	134 (53.8%)
**What type of publication do you currently work for?***	
Print	50
Online	105
Daily	27
Weekly	21
Monthly	11
Other	1

table S7. Results from the principal component analysis and meaning extraction method for *The Washington Post*. Components were rotated using the varimax method. λ = eigenvalues. % = amount of variance explained by each component. For this analysis, unigrams (single words), bigrams (two-word phrases), and trigrams (three-word phrases) were all extracted as possible terms for meaning extraction.

Component 1: Today		Component 2: Ukraine War		Component 3: Climate Change		Component 4: White House		Component 5: COVID-19	
λ	%	λ	%	λ	%	λ	%	λ	%
1.67	4.39	1.49	3.91	1.48	3.91	1.48	3.90	1.15	3.03
Word	Loading	Word	Loading	Word	Loading	Word	Loading	Word	Loading
things	0.889	Ukraine	0.697	change	0.857	white	0.859	China	0.720
today	0.889	war	0.646	climate	0.857	house	0.855	Covid	0.701
		Putin	0.526						
		Russia	0.525						

table S8. Results from the principal component analysis and meaning extraction method for *Upworthy*. Components were rotated using the varimax method. λ = eigenvalues. % = amount of variance explained by each component. For this analysis, unigrams (single words), bigrams (two-word phrases), and trigrams (three-word phrases) were all extracted as possible terms for meaning extraction.

Component 1: Race/Ethnicity		Component 2: Questions		Component 3: Gender		Component 4: Videos		Component 5: Problems	
λ	%	λ	%	λ	%	λ	%	λ	%
1.35	2.20	1.26	2.07	1.23	2.01	1.14	1.87	1.13	1.86
Word	Loading	Word	Loading	Word	Loading	Word	Loading	Word	Loading
White	0.710	question	0.777	women	0.749	watch	0.676	problem	0.666
Black	0.703	asked	0.775	men	0.718	video	0.616	big	0.628
people	0.392								
man	0.326								

REFERENCES AND NOTES

1. S. Vosoughi, D. Roy, S. Aral, The spread of true and false news online, *Science* **359**, 1146–1151 (2018).

2. J. Allen, B. Howland, M. Mobius, D. Rothschild, D. J. Watts, Evaluating the fake news problem at the scale of the information ecosystem *Sci. Adv.* **6**, eaay3539 (2020)

3. V. Bélair-Gagnon, L. Graves, B. Kalsnes, S. Steensen, O. Westlund, Considering interinstitutional visibilities in combating misinformation. *Digit. Journal.* **10**, 669, 678 (2022).

4. J. F. Sparks, J. D. Hmielowski, At the extremes: Assessing readability, grade level, sentiment, and tone in US media outlets, *Journal. Stud.* **24**, 24–44 (2023).

5. A. M. Guess, P. Barberá, B. Barberá, S. Munzert, J. Yang, The consequences of online partisan media, *Proc. Natl. Acad. Sci. U.S.A.* **118**, e2013464118 (2021).

6. M. Carlson, The information politics of journalism in a post-truth age. *Journal. Stud.* **19**, 1879–1888 (2018).

7. S. T. Fiske, S. E. Taylor, *Social Cognition* (Mcgraw-Hill Company, ed. 2nd, 1991).

8. P. Pirolli, S. Card, Information foraging, *Psychol. Rev.* **106**, 643–675 (1999).

9. T. Rogers, J. Lasky-Fink, *Writing for busy readers: Communicate more effectively in the real world* (Penguin, 2023).

10. T. P. Vos, R. J. Thomas, “They’re making it more democratic”: The normative construction of participatory journalism. *Digit. Journal.* **1-25** (2023), 1, 25.

11. E. Martínez, F. I. Mollica, E. Gibson, Even lawyers do not like legalese, *Proc. Natl. Acad. Sci. U.S.A.* **120**, e2302672120 (2023).

12. H. C. Shulman, G. N. Dixon, O. M. Bullock, D. Colón Amill, The effects of jargon on processing fluency, self-perceptions, and scientific engagement. *J. Lang. Soc. Psychol.* **39**, 579–597 (2020).

13. P. Tolochko, H. Song, H. Boomgaarden, "That looks hard!": Effects of objective and perceived textual complexity on factual and structural political knowledge. *Polit. Commun.* **36**, 609–628 (2019).

14. D. M. Markowitz, H. C. Shulman, The predictive utility of word familiarity for online engagements and funding. *Proc. Natl. Acad. Sci. U.S.A.* **118**, e2026045118 (2021).

15. C. E. Robertson, N. Pröllochs, K. Schwarzenegger, P. Pärnamets, J. J. Van Bavel, S. Feuerriegel, Negativity drives online news consumption, *Nat. Hum. Behav.* **7**, 812–822 (2023).

16. A. Fishbane, A. Ouss, A. K. Shah, Behavioral nudges reduce failure to appear for court. *Science* **370**, eabb6591 (2020).

17. J. R. Kling, S. Mullainathan, E. Shafir, L. C. Vermeulen, M. V. Wrobel, Comparison friction: Experimental evidence from Medicare drug plans. *Q. J. Econ.* **127**, 199–235 (2012).

18. E. P. Bettinger, B. T. Long, P. Oreopoulos, L. Sanbonmatsu, The role of application assistance and information in college decisions: Results from the H&R Block Fafsa experiment, *Q. J. Econ.* **127**, 1205–1242 (2012).

19. H. Stanislaw, N. Todorov, Calculation of signal detection theory measures. *Behav. Res. Methods. Instrum. Comput.* **31**, 137–149 (1999).

20. P. J. Hinds, M. Patterson, J. Pfeffer, Bothered by abstraction: The effect of expertise on knowledge transfer and subsequent novice performance. *J. Appl. Psychol.* **86**, 1232–1243 (2001).

21. P. Biyani, K. Tsioutsiouliklis, J. Blackmer, "'8 Amazing Secrets for Getting More Clicks': Detecting Clickbaits in News Streams Using Article Informality"; http://news.yahoo.com/justin-bieber-makes-huge-life-.

22. D. M. Oppenheimer, Consequences of erudite vernacular utilized irrespective of necessity: Problems with using long words needlessly, *Applied Cognitive Psychology* **20**, 139–156 (2006).

23. H. C. Shulman, M. D. Sweitzer, Advancing framing theory: Designing an equivalency frame to improve political information processing. *Hum. Commun. Res.* **44**, 155–175 (2018).

24. M. D. Sweitzer, H. C. Shulman, The effects of metacognition in survey research: Experimental, cross-sectional, and content-analytic evidence. *Public Opin. Q.* **82**, 745–768 (2018).

25. H. C. Shulman, M. D. Sweitzer, Varying metacognition through public opinion questions: How language can affect political engagement. *J. Lang. Soc. Psychol.* **37**, 224–237 (2018).

26. Guide to headline testing and image testing, *Chartbeat* (2023).

27. J. W. Pennebaker, R. L. Boyd, R. J. Booth, A. Ashokkumar, M. E. Francis, *Linguistic inquiry and word count: LIWC-22*. Pennebaker Conglomerates (2022).

28. R. L. Boyd, H. A. Schwartz, Natural language analysis and the psychology of verbal behavior: The past, present, and future states of the field, *J. Lang. Soc. Psychol.* **40**, 21–41 (2021).

29. D. M. Markowitz, Instrumental goal activation increases online petition support across languages. *J. Pers. Soc. Psychol.* **124,** 1133–1145 (2023).

30. WashPostPR, The Washington Post records more than 71 million unique digital visitors in December 2021, *The Washington Post* (2022). https://www.washingtonpost.com/pr/2022/01/25/washington-post-records-more-than-71-million-unique-digital-visitors-december-2021/.

31. A. D. I. Kramer, J. E. Guillory, J. T. Hancock, Experimental evidence of massive-scale emotional contagion through social networks. *Proc. Natl. Acad. Sci. U.S.A.* **111**, 8788–8790 (2014).

32. A. Gallo, A refresher on A/B testing, *Harvard Business Review*, 28 June 2017.

33. D. M. Markowitz, J. T. Hancock, Linguistic obfuscation in fraudulent science. *J. Lang. Soc. Psychol.* **35**, 435–445 (2016).

34. Y. R. Tausczik, J. W. Pennebaker, *The psychological meaning of words: LIWC and computerized text analysis methods*. *J. Lang. Soc. Psychol.* **29**, 24–54 (2010).

35. J. W. Pennebaker, C. K. Chung, J. Frazee, G. M. Lavergne, D. I. Beaver, When small words foretell academic success: The case of college admissions essays. *PLOS ONE* **9**, e115844 (2014).

36. R. Flesch, A new readability yardstick. *J. Appl. Psychol.* **32**, 221–233 (1948).

37. K. Benoit, K. Watanabe, H. Wang, P. Nulty, A. Obeng, S. Müller, A. Matsuo, quanteda: An R package for the quantitative analysis of textual data. *J. Open Source Softw.* **3**, 774 (2018).

38. J. N. Matias, K. Munger, M. A. Le Quere, C. Ebersole, The Upworthy Research Archive, a time series of 32,487 experiments in U.S. media. *Sci Data* **8**, 195 (2021),.

39. L. Litman, J. Robinson, T. Abberbock, TurkPrime.com: A versatile crowdsourcing data acquisition platform for the behavioral sciences. *Behav. Res. Methods.* **49**, 433–442 (2017).

40. H. C. Shulman, O. M. Bullock, Don't dumb it down: The effects of jargon in COVID-19 crisis communication. *PLOS ONE* **15**, e0239524 (2020).

41. H. C. Shulman, O. M. Bullock, E. E. Riggs, The interplay of jargon, motivation, and fatigue while processing COVID-19 crisis communication over time, *J. Lang. Soc. Psychol.* **40**, 546–573 (2021).

42. R. E. Petty, J. T. Cacioppo, "The elaboration likelihood model of persuasion" in *Advances in Experimental Social Psychology*, L. Berkowitz, Ed. (Academic Press, New York, 1986; 19, pp. 123–205).

43. K. Gligorić, G. Lifchits, R. West, A. Anderson, Linguistic effects on news headline success: Evidence from thousands of online field experiments (Registered Report). *PLOS ONE* **18,** e0281682 (2023).

44. D. Bates, M. Mächler, E. Zurich, B. M. Bolker, S. C. Walker, Fitting linear mixed-effects models using lme4. *J. Stat. Softw.* **67**, 1–48 (2015).

45. A. Kuznetsova, P. B. Brockhoff, R. H. B. Christensen, lmerTest package: Tests in linear mixed effects models. *J. Stat. Softw.* **82**, 1–26 (2017).

46. C. K. Chung, J. W. Pennebaker, Revealing dimensions of thinking in open-ended self-descriptions: An automated meaning extraction method for natural language, *J. Res. Pract.* **42**, 96–132 (2008).

47. D. M. Markowitz, The meaning extraction method: An approach to evaluate content patterns from large-scale language data. *Front. Commun.* **6** (2021).

48. D. M. Blei, A. Y. Ng, M. I. Jordan, "*Latent Dirichlet Allocation*" (2002).

49. D. M. Markowitz, Self-presentation in medicine: How language patterns reflect physician impression management goals and affect perceptions. *Comput. Human Behav.* **143**, 107684 (2023).

50. D. M. Markowitz, M. Kouchaki, J. T. Hancock, F. Gino, The deception spiral: Corporate obfuscation leads to perceptions of immorality and cheating behavior, *J. Lang. Soc. Psychol.* **40**, 277–296 (2021).

51. M. Chmielewski, S. C. Kucker, An MTurk crisis? Shifts in data quality and the impact on study results. **11**, 464–473 (2020).
